# Supplementary material for: Real‐Time Monitoring of DNA Origami‐Cell Interactions via Single Particle Tracking
Source: Small. 2025 Jun 30;21(40):2502496. doi: 10.1002/smll.202502496 (PMC12508699; doi:10.1002/smll.202502496)
Supplement: Supplementary file 1 — Supporting Information [file SMLL-21-2502496-s001.docx]

Supporting Information

Real-Time Monitoring of DNA Origami-Cell Interactions via Single Particle Tracking

Indra Van Zundert^‡^, Elena Spezzani^‡^, Roger R. Brillas, Lars Paffen, Angelina Yurchenko, Tom F. A. de Greef, Lorenzo Albertazzi, Alessandro Bertucci and Tania Patiño*

^‡^ These authors contributed equally

**Supplementary Table S1.** Anti-handle for Anti-EGFR Antibody/Aptamer labeling.

This 3’azide-oligo was used for NHS coupling to the Anti-EGFR Antibody or Aptamer.

| **ID** | **Sequence (5’ to 3’)** |
| --- | --- |
| L | N3 - CGACGACGACGACGACGACGA |

**Supplementary Table S2.** Fluorescently labeled imagers

To label DNA nanostructures, complementary imager (F) strands were used during the self-assembly of the DNA nanostructure.

| **ID** | **Sequences (5’ to 3’)** |
| --- | --- |
| F | CAGTCAGTCAGTCAGTCAGT-Atto647 |

**Supplementary Table S3.** Handle-extended staple strands antibody incorporation.

To assemble DNA nanorods for anti-EGFR Antibody/Aptamer, appropriate unmodified staples were replaced with handle-extended staple strands. Bold nucleotides refer to the extended handle sequence. Labels: A, antibody/aptamer; C, fluorophore.

| **ID** | **Unmodified Staple ID** | **Used-anti handle** | **Sequences (5’ to 3’)** |
| --- | --- | --- | --- |
| mC4.1 | Oligo 14 | F | **ACTGACTGACTGACTGACTG**TTTGCTGCATTGTAAACGTTAATTAGAACC |
| mC4.2 | Oligo 15 | F | **ACTGACTGACTGACTGACTG**TTCTCATATATAAGAGGTCATTTAGTTTTG |
| mC4.3 | Oligo 16 | F | **ACTGACTGACTGACTGACTG**TTCCAGAGGAAGAGTATTTTTCATGAGGAATCCACAG |
| mC5.1 | Oligo 17 | F | **ACTGACTGACTGACTGACTG**TTGCGGGCCGTTAAATCAGCTCATTGCGGG |
| mC5.2 | Oligo 18 | F | **ACTGACTGACTGACTGACTG**TTAGAAGCCAATATAATGCTGTAACGACGA |
| mC5.3 | Oligo 19 | F | **ACTGACTGACTGACTGACTG**TTTAAAAACAACGTAAACGAGGGTAGCAACTGTCGTC |
| AmM1 | Oligo 68 | L | GCCTGAGTAGAATTTCCCACCGAGTAAAAGAGTCTATCACTTTT**TCGTCGTCGTCGTCGTCGTCG** |
| AmM2 | Oligo 72 | L | GCCACCACGTCGAGAGGGTTGAATTAGAGCCAGCACAAAAGGTT**TCGTCGTCGTCGTCGTCGTCG** |
| AmM3 | Oligo 75 | L | CCATGTATAGGATTAGCGGGGCAAGGCCGGAAACGCACAATCTT**TCGTCGTCGTCGTCGTCGTCG** |
| AmM4 | Oligo 78 | L | AACGCCTACATGAAAGTATTACGTAATCAGTAGCGATAAAAGTT**TCGTCGTCGTCGTCGTCGTCG** |
| AmM5 | Oligo 84 | L | ATTTTCTTTTTAACGGGGTCATATTAGCGTTTGCCCCCAAAATT**TCGTCGTCGTCGTCGTCGTCG** |
| AmM6 | Oligo 87 | L | AGCGGAGCATACATGGCTTTTCAGAGCCACCACCGGTTACCATT**TCGTCGTCGTCGTCGTCGTCG** |
| AmM7 | Oligo 90 | L | TAATAATAATGGAAAGCGCAGCAGAACCGCCACCCCCGAAGCTT**TCGTCGTCGTCGTCGTCGTCG** |
| AmM8 | Oligo 93 | L | AAAAGGAGGCCTTGATATTCACAGAACCACCACCAATAATAATTTCG**TCGTCGTCGTCGTCGTCG** |
| AmR1 | Oligo 20 | L | CCGATTTGAGAAAGGAAGGGAGCGCGTATT**TCGTCGTCGTCGTCGTCGTCG** |
| AmR2 | Oligo 22 | L | TTAGTGCAGGCTATCAGGTCATTTTTGATT**TCGTCGTCGTCGTCGTCGTCG** |
| AmR3 | Oligo 23 | L | CTTCTAAAATCGATGAACGGTCAACCGTTT**TCGTCGTCGTCGTCGTCGTCG** |
| AmR4 | Oligo 24 | L | GGCCAGTGTACCCCGGTTGATGAGAAAGTT**TCGTCGTCGTCGTCGTCGTCG** |
| AmR5 | Oligo 25 | L | GGTAACGTTGTATAAGCAAATATGCAATTT**TCGTCGTCGTCGTCGTCGTCG** |
| AmR6 | Oligo 26 | L | CCAGCTGTAAAATTCGCATTACAACGCATT**TCGTCGTCGTCGTCGTCGTCG** |
| AmR7 | Oligo 27 | L | CTGTTGGACCAATAGGAACGCCATTATGTT**TCGTCGTCGTCGTCGTCGTCG** |
| AmR8 | Oligo 28 | L | CGGAAACCTGTAGCCAGCTTTATAAAGCTT**TCGTCGTCGTCGTCGTCGTCG** |
| AmR9 | Oligo 29 | L | AAGATCGACCCGTCGGATTCTATAAATCTT**TCGTCGTCGTCGTCGTCGTCG** |
| AmR10 | Oligo 30 | L | TAGGTCAAGGTGGCATCAATTCTGTTTAGCTATATACGAACTTT**TCGTCGTCGTCGTCGTCGTCG** |

**Supplementary Table S4.** Sequences of unmodified staple strands of the DNA nanorod.

Locations of the 5’ and 3’ end are indicated using the reference helix number, with the reference nucleotide position denoted in brackets.

| **Staple ID** | **Location of 5' end** | **Location of 3' end** | **Sequence** |
| --- | --- | --- | --- |
| 1 | 0[51] | 14[42] | AAGACACCGCCTAACTGGCGCGGTAAGCCAACAGAGAT |
| 2 | 0[93] | 14[94] | GGAGAAAAATAACAGTACTTGAAACAAG |
| 3 | 0[114] | 17[104] | GGTTGCTGAATGAATTACCTTTTTTAATGGACTAAAGC |
| 4 | 0[135] | 14[136] | CAACCCTCAAATTACATGTCAATAAGAA |
| 5 | 0[156] | 17[146] | TCGGTTGGCAAACATCAAGAAAACAAAATTATCAATAT |
| 6 | 0[177] | 14[178] | TGTAGGAATTGCAAAAGCTTTTTATAGA |
| 7 | 0[198] | 17[188] | TGCAAAATATTTATTCATTTCAATTACCTGAGAGGAAG |
| 8 | 0[219] | 14[220] | AGCACAACTAACAAAATAAATGCTCTGA |
| 9 | 0[240] | 17[230] | GCCTCAATAGGATTGCTTTGAATACCAAGTTATAGATT |
| 10 | 0[261] | 14[262] | AACGATTTAGGAAACAAAAACTTTAGCT |
| 11 | 0[282] | 17[272] | CTTACAAACAACAGTACCTTTTACATCGGGAAAGTATT |
| 12 | 0[303] | 14[304] | TTGATTAAATACGTCAGTAAATTTTAAA |
| 13 | 0[324] | 17[314] | CGGTTATTAATTGCGTAGATTTTCAGGTTTACCTTTGC |
| 14 | 0[345] | 14[346] | ATTGTAACATCGTAAAACGTTAAATTTC |
| 15 | 0[366] | 17[356] | GAAACAAAGAACCATATCAAAATTATTTGCATATCATT |
| 16 | 0[387] | 14[388] | CACGCGGAATATAATGGAGCCTGTGCCA |
| 17 | 0[408] | 17[398] | TCATGATTATTTGTTTGGATTATACTTCTGATATCATC |
| 18 | 0[419] | 3[412] | AGTGAGCCATACGAAACCGTGCATCTGCAATGGGA |
| 19 | 1[73] | 17[58] | TCAATTAAAGACGCTGAGTGTGAGTGAATAACCTTGCTTCTG |
| 20 | 2[34] | 4[35] | CCGATTTGAGAAAGGAAGGGAGCGCGTA |
| 21 | 2[55] | 2[56] | TCGGAACGAAAGGAGCGGGCGCTAGGAATGTAAAGCACTAAA |
| 22 | 2[76] | 4[77] | TTAGTGCAGGCTATCAGGTCATTTTTGA |
| 23 | 2[118] | 4[119] | CTTCTAAAATCGATGAACGGTCAACCGT |
| 24 | 2[160] | 4[161] | GGCCAGTGTACCCCGGTTGATGAGAAAG |
| 25 | 2[202] | 4[203] | GGTAACGTTGTATAAGCAAATATGCAAT |
| 26 | 2[244] | 4[245] | CCAGCTGTAAAATTCGCATTACAACGCA |
| 27 | 2[286] | 4[287] | CTGTTGGACCAATAGGAACGCCATTATG |
| 28 | 2[328] | 4[329] | CGGAAACCTGTAGCCAGCTTTATAAAGC |
| 29 | 2[370] | 4[371] | AAGATCGACCCGTCGGATTCTATAAATC |
| 30 | 3[413] | 6[413] | TAGGTCAAGGTGGCATCAATTCTGTTTAGCTATATACGAACT |
| 31 | 4[34] | 7[34] | ACCACCAGCGTACTATGGTTGAAACAGGAGGCCGAGAATCCT |
| 32 | 4[55] | 4[56] | GTAGCGGGAGCACGTATAACGTGCTTTCACGCGCTGGCAAGT |
| 33 | 4[76] | 6[77] | GAGATCTAGAAGCAAAGCGGAACCCTGA |
| 34 | 4[107] | 0[115] | AATACAAGAGGTGGTTGCCCGCTTCTAATCTATAG |
| 35 | 4[118] | 6[119] | TCTAGCTGAAAGACTTCAAATTTCAGAA |
| 36 | 4[149] | 0[157] | TCAATCATATGCCAAGCGATACCGACAGTGCGAAA |
| 37 | 4[160] | 6[161] | GCCGGAGAACCAGACCGGAAGGTCATAA |
| 38 | 4[191] | 0[199] | GTGAGGAAGACCAGGGTGTGGGCACGAATATGGTT |
| 39 | 4[202] | 6[203] | GCCTGAGTACCTTTAATTGCTAGTAAAA |
| 40 | 4[233] | 0[241] | TTTATTTTGTGCGAAAGACATAAATCATTTCCACC |
| 41 | 4[244] | 6[245] | AGGATAATGGCTTAGAGCTTAGCGAGAG |
| 42 | 4[275] | 0[283] | CTTTTTTTTAGAAGGGCATGAGTAAACAGGGTTTT |
| 43 | 4[286] | 6[287] | ACCCTGTATGTTTTAAATATGATCATAA |
| 44 | 4[317] | 0[325] | AAGGGCCTTCCAGGCAAATAAAGACGGAGGACGCG |
| 45 | 4[328] | 6[329] | CTCAGAGATTCCATATAACAGATAACGC |
| 46 | 4[359] | 0[367] | GCAAGTAACACACTCCATCATGGTCATAGCTTCGG |
| 47 | 4[370] | 6[371] | ATACAGGTTTAGTTTGACCATCAGTTGA |
| 48 | 4[401] | 0[409] | TAATGACCGTCAGTTTGACAATTCCACACAATAAC |
| 49 | 5[46] | 0[52] | GACTCACGCTAGAAAGCCCTAAAGTCAAGTTTTTTGGGGTCA |
| 50 | 5[88] | 0[94] | ATCGTAGCTATTGCCTGCGACTTAACAATGTCCCGCCAGTTT |
| 51 | 5[130] | 0[136] | CGTTGATATTAATCGTACCAGGGTCTCGCCCTGGAGTGAAAT |
| 52 | 5[172] | 0[178] | CTCAAAGGGTAATCAGACGTTGTACCATCTGTAAGCAAATCC |
| 53 | 5[214] | 0[220] | TTGATTTTAAATTTAAAAGGCGATTTGAATCGGCTGACTTGC |


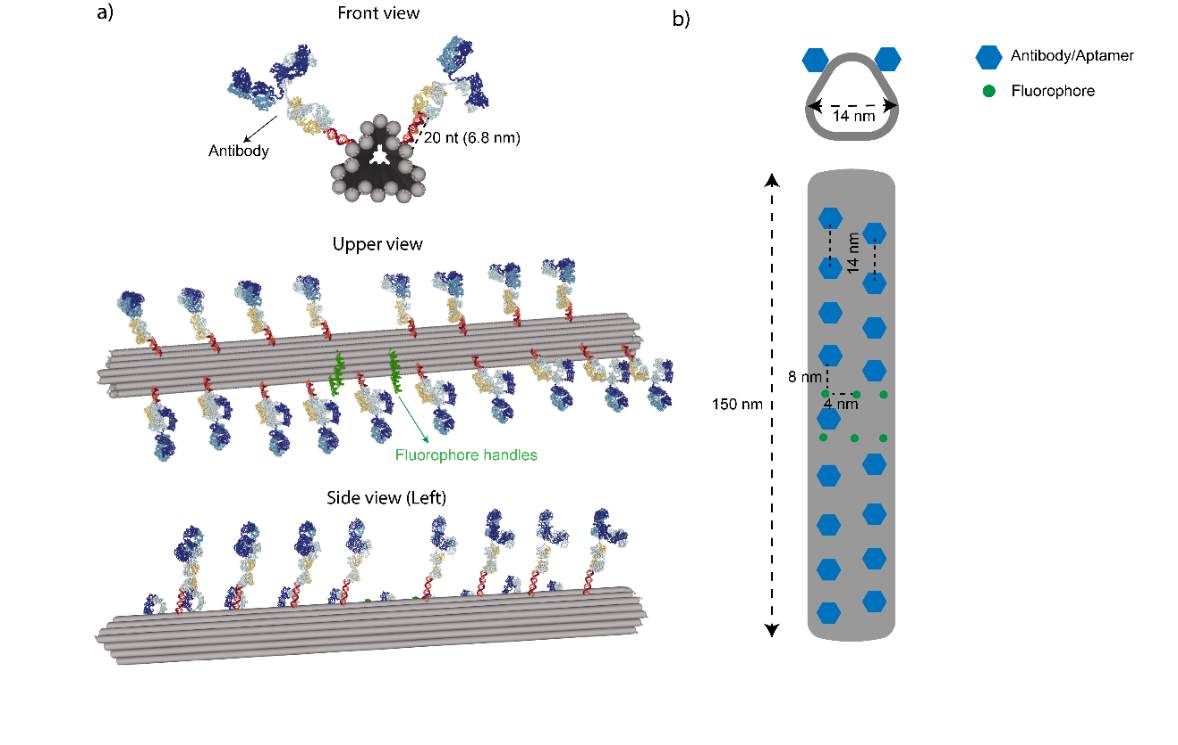


**Figure S1:** Illustrative scheme of the nanorod design. a) Realistic front, upper and left view on the nanorod structure made with the open source Catana software. In this render, the antibodies are conjugated to the nanorod, however, the aptamers would be located in the same positions. The 6 positions of the fluorophores are depicted in green. b) Conceptual representation of the nanorod design with the location of the antibody/aptamer in blue and the locations of the fluorophore in green.


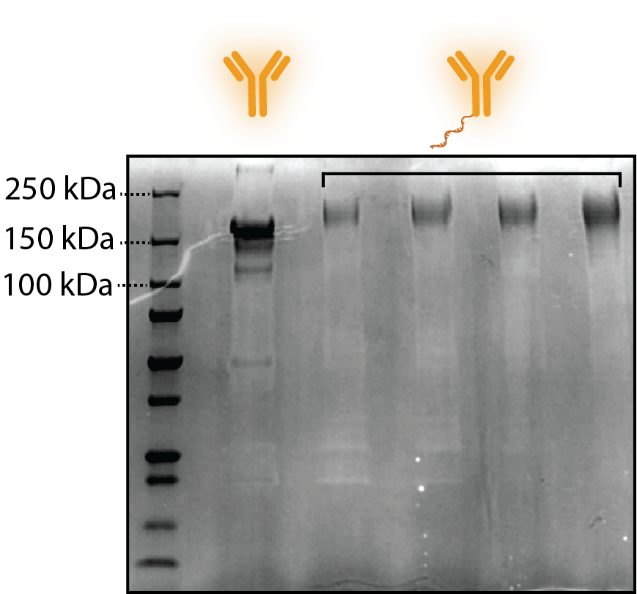


**Figure S2.** Non-reducing SDS page gel of the EGFR antibody (first lane, 2µM) and the oligo conjugated EGFR antibody (lane 2-5, increasing concentration of antibody, 120 nM, 240 nM, 600 nM and 1.2 2µM)


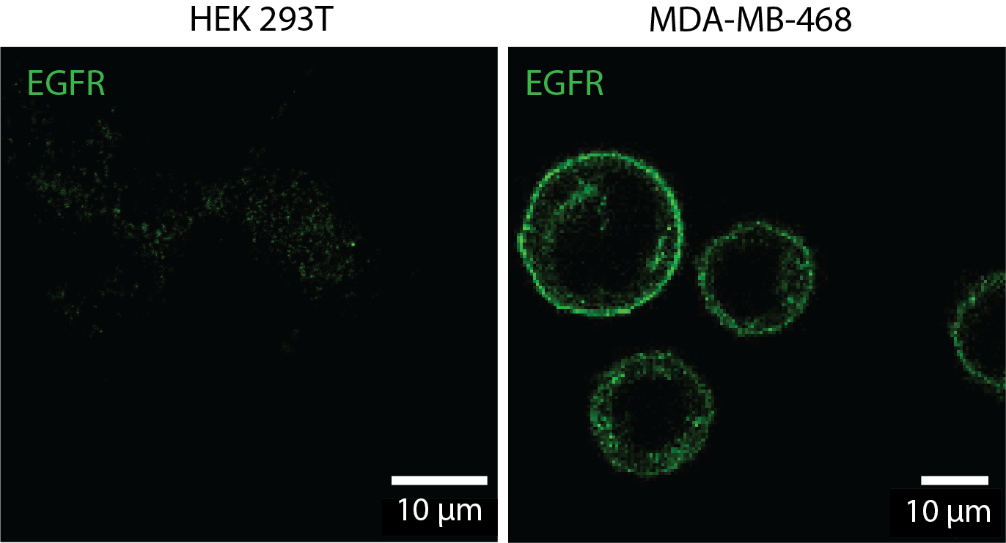


µm

µm

**Figure S3:** Immunostaining of the EGFR on HEK 293T cells (left) and the MDA-MB-468 cells (right). Images were taken via confocal laser scanning microscopy.

_
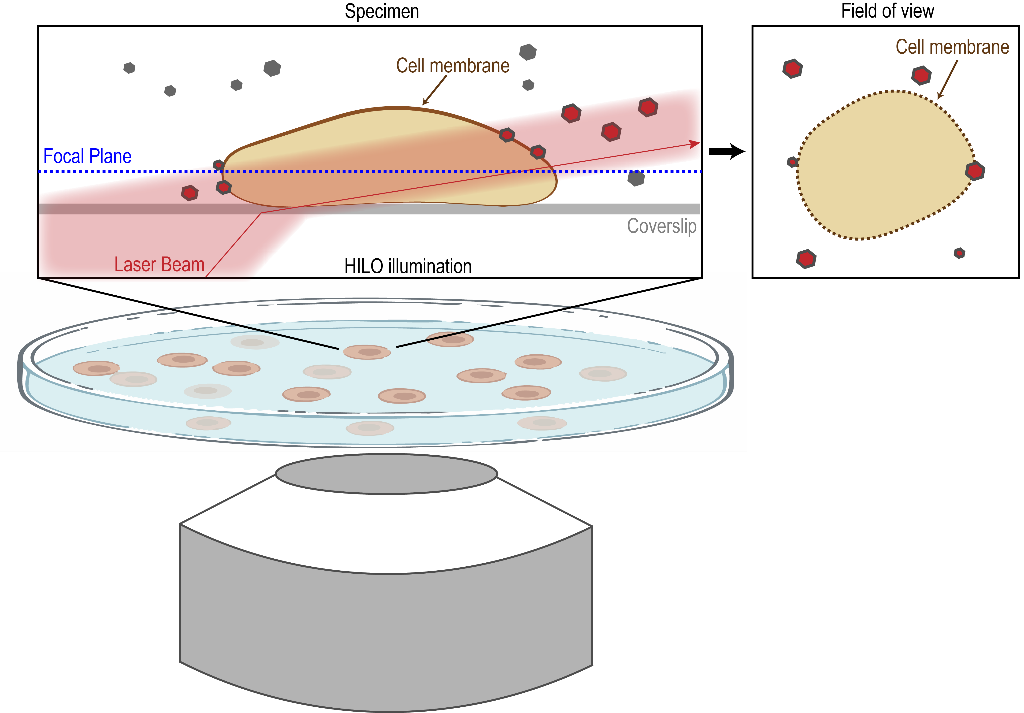
_

**Figure S4:** Schematic sketch of the single particle tracking experiments in cells. Illumination is performed with highly inclined and laminated optical sheet (HiLo) microscopy, enhancing the single to noise ratio compared to standard wide field EPI illumination.


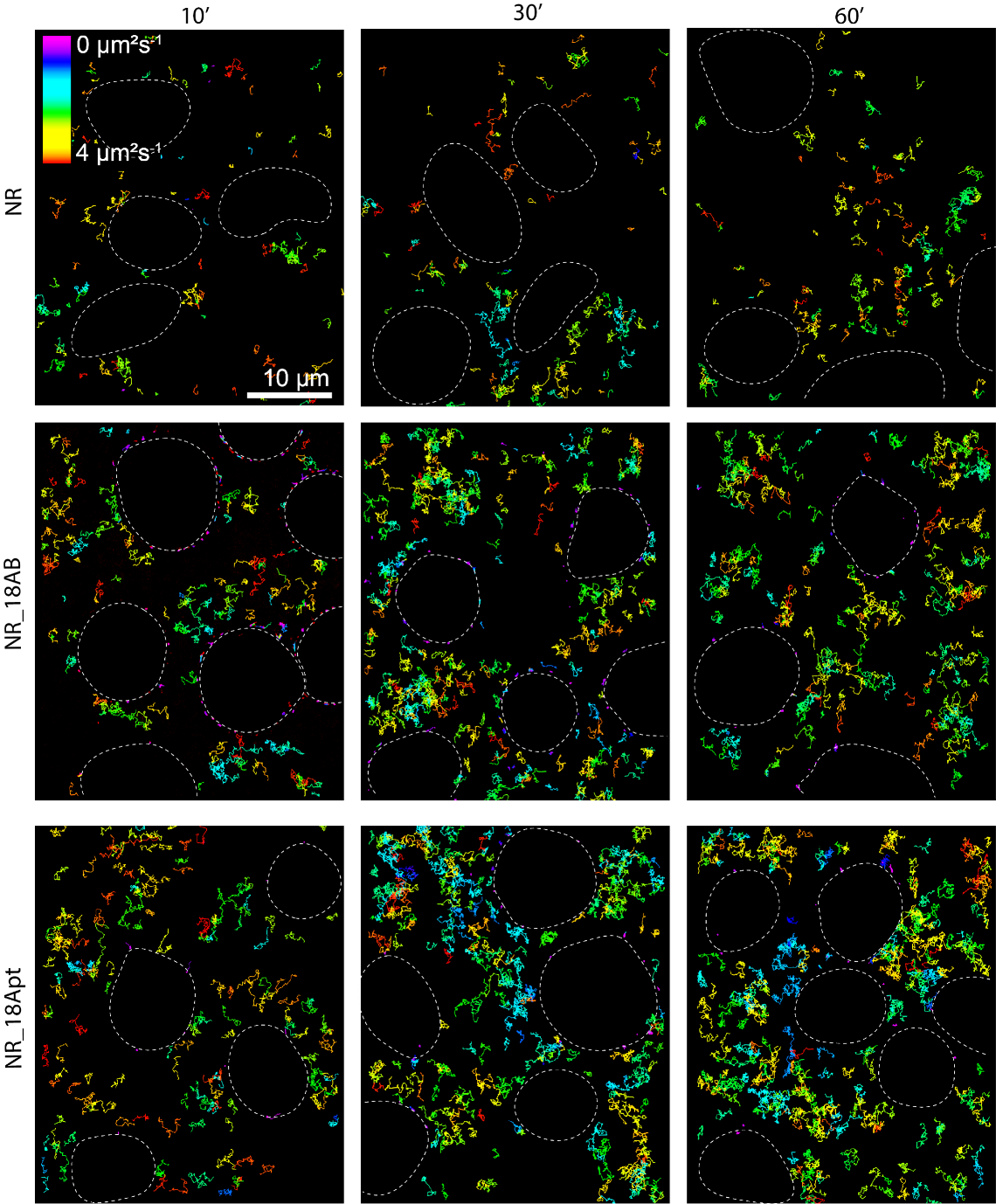


**Figure S5:** Plots of the trajectories of the different NR designs (empty NR, NR_18Ab and NR_18Apt) 10, 30 and 60 min after NR administration to MDA-MB-468 cells (1 representative trajectory plot per condition). The cell contours are indicated by the dotted line. Scale bars values are displayed on the image, the color bar represents variations in diffusion coefficients (ranging from 0 to 4 µm2/s). Scale bar is 10 µm.
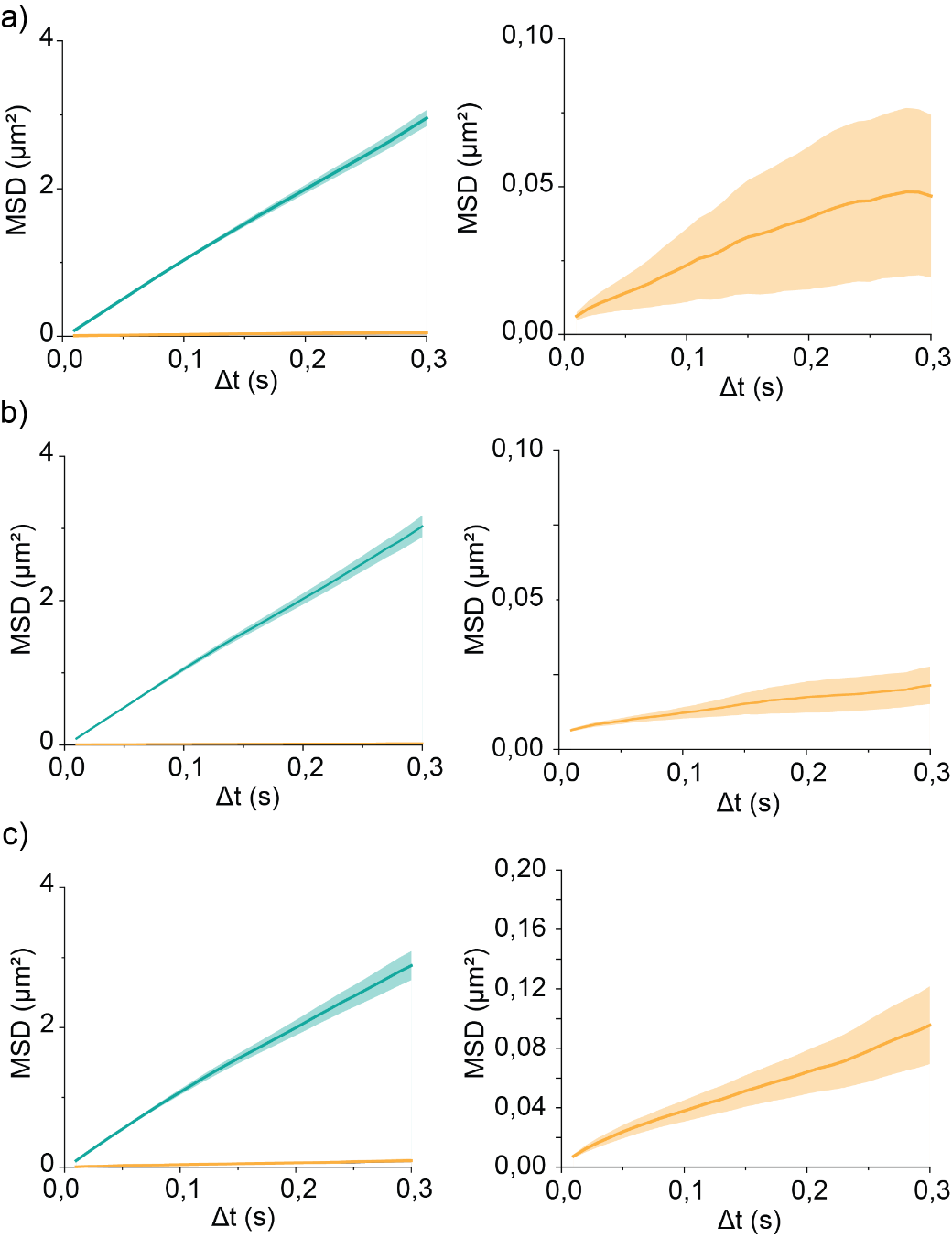


**Figure S6.** Plots of the mean squared displacement over time for a) the empty NR, b) NR_18Ab and c) NR_18Apt. Trajectories that have a diffusion coefficient higher than 1 (free diffusing NRs) are plotted in blue whereas trajectories with a diffusion coefficient lower or equal to 1 (receptor binding NRs) are plotted in yellow. Since the receptor binding NRs have a very low mobility, the yellow line is located too close to the x-axis. Therefore, the axes were rescaled to clarify the trend of the receptor binding NR population (second column).


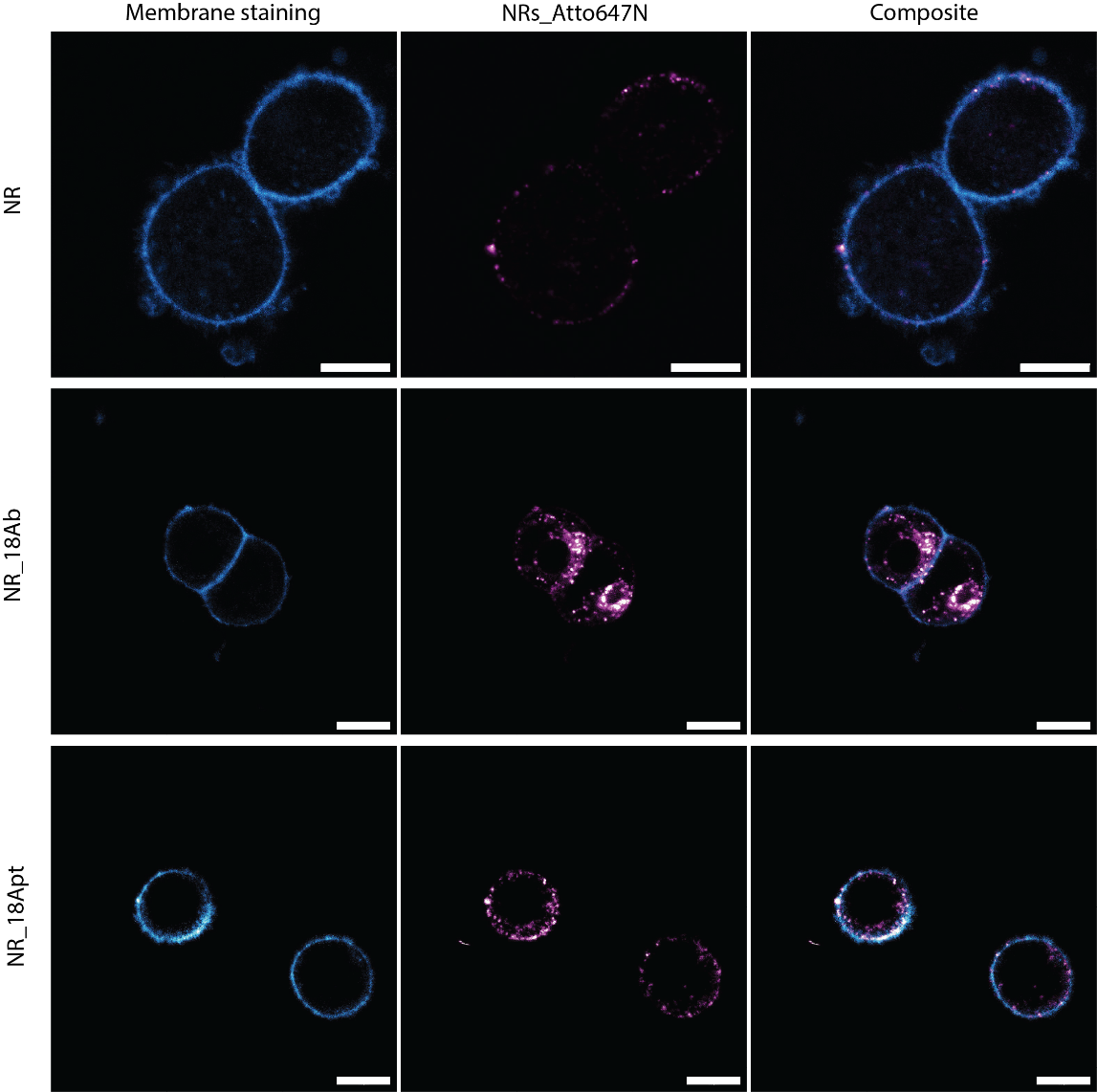


**Figure S7.** Confocal laser scanning microscopy images of MDA-MB-468 cells incubated with empty NRs, NRs_18Ab and NR_18Apt, all labeled with Atto648N (magenta). The membrane is stained with BioTracker™ 400 Blue Cytoplasmic Membrane Dye (cyan). Scale bar is 10 µm.


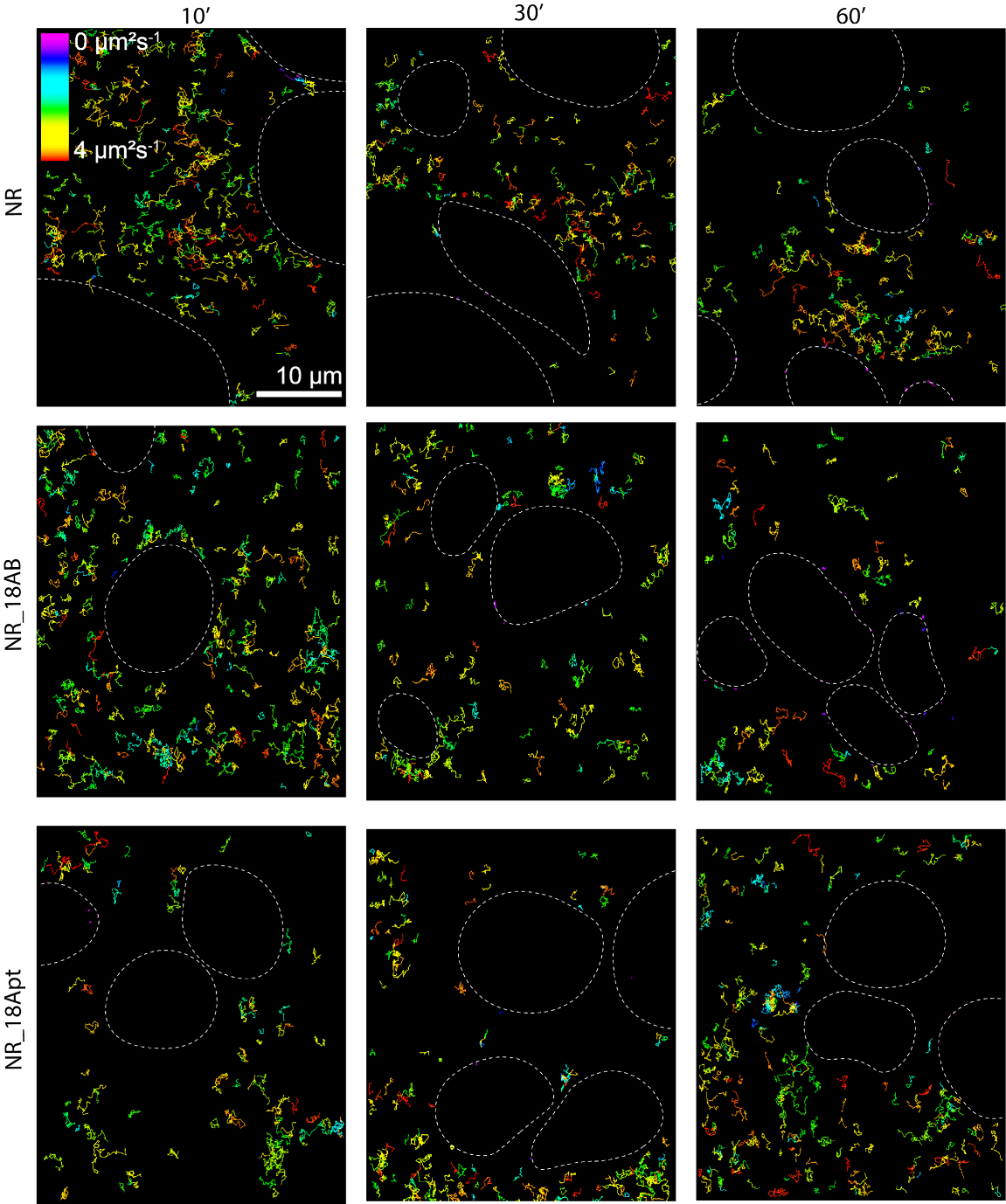


**Figure S8:** Plots of the trajectories of the different NR designs (empty NR, NR_18Ab and NR_18Apt) 10, 30 and 60 min after NR administration to HEK 293T cells (1 representative trajectory plot per condition). The cell contours are indicated by the dotted line. Scale bars values are displayed on the image, the color bar represents variations in diffusion coefficients (ranging from 0 to 4 µm2/s). Scale bar is 10 µm.


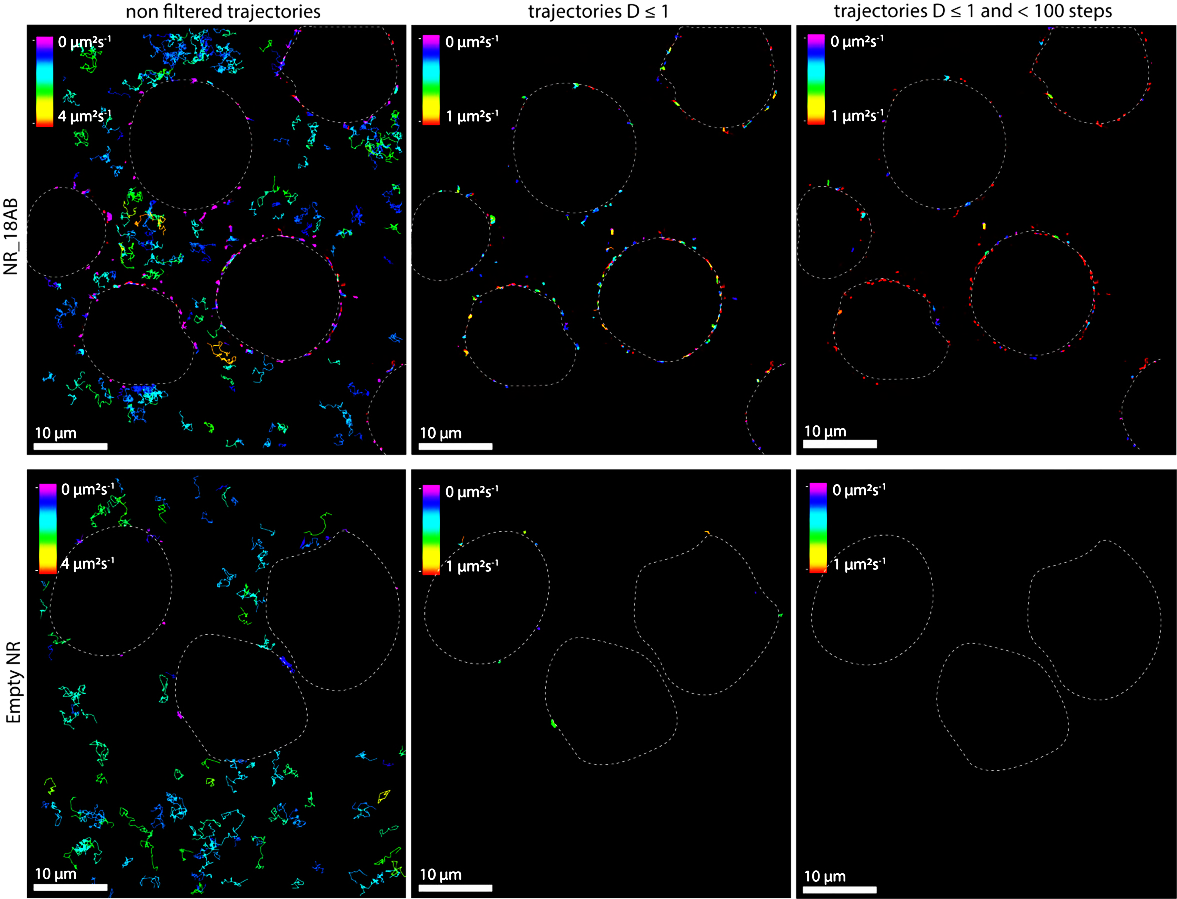


**Figure S9:** Representative illustration of the trajectories from NR_18AB (upper panels) and the empty NR (bottom panels) before and after the application of the criteria for binding (D ≤ 1) and specific binding (D ≤ 1 and the trajectory is longer than 100 steps).


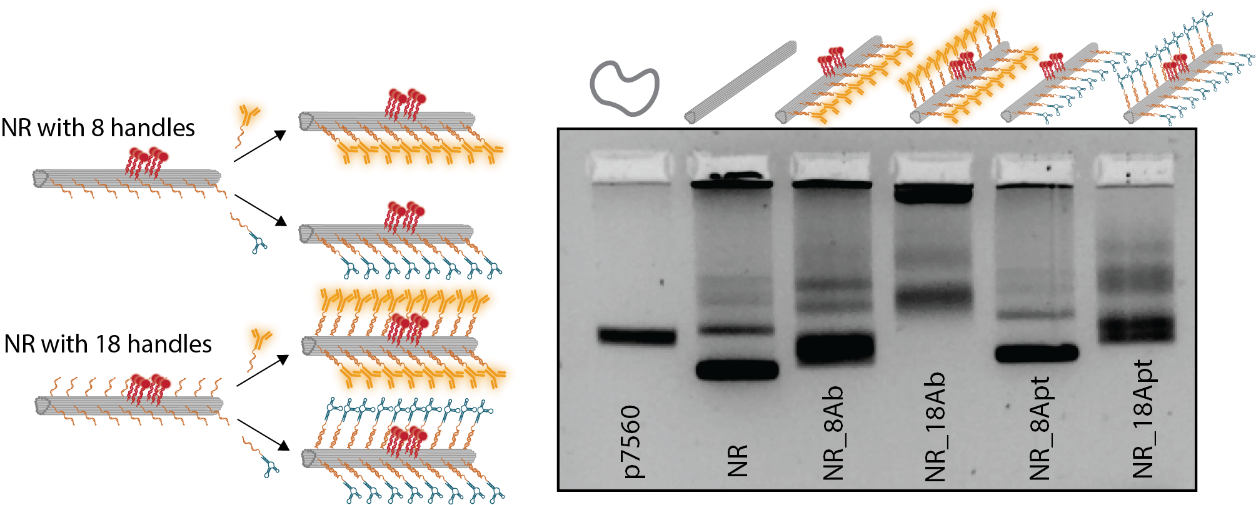


**Figure S10**. Agarose gel of nanorods conjugated with 8 or 18 EGFR antibodies and 8 or 18 aptamers.
